# Individual versus superensemble forecasts of seasonal influenza outbreaks in the United States

**DOI:** 10.1371/journal.pcbi.1005801

**Published:** 2017-11-06

**Authors:** Teresa K. Yamana, Sasikiran Kandula, Jeffrey Shaman

**Affiliations:** Department of Environmental Health Sciences, Mailman School of Public Health, Columbia University, New York, New York, United States of America; Johns Hopkins Bloomberg School of Public Health, UNITED STATES

## Abstract

Recent research has produced a number of methods for forecasting seasonal influenza outbreaks. However, differences among the predicted outcomes of competing forecast methods can limit their use in decision-making. Here, we present a method for reconciling these differences using Bayesian model averaging. We generated retrospective forecasts of peak timing, peak incidence, and total incidence for seasonal influenza outbreaks in 48 states and 95 cities using 21 distinct forecast methods, and combined these individual forecasts to create weighted-average superensemble forecasts. We compared the relative performance of these individual and superensemble forecast methods by geographic location, timing of forecast, and influenza season. We find that, overall, the superensemble forecasts are more accurate than any individual forecast method and less prone to producing a poor forecast. Furthermore, we find that these advantages increase when the superensemble weights are stratified according to the characteristics of the forecast or geographic location. These findings indicate that different competing influenza prediction systems can be combined into a single more accurate forecast product for operational delivery in real time.

## Author summary

Timely forecasts of infectious disease transmission can help public health officials, health care providers, and individuals better prepare for and respond to disease outbreaks. Work in recent years has led to the development of a number of forecast systems. These systems provide important information on future disease incidence; however, all forecasting systems contain inaccuracies, or error. This error can be reduced by combining information from multiple forecasting systems into a superensemble using Bayesian averaging methods. Here we compare 21 forecasting systems for seasonal influenza outbreaks and use them together to create superensemble forecasts. The superensemble produces more accurate forecasts than the individual systems, improving our ability to predict the timing and severity of seasonal influenza outbreaks.

## Introduction

In the United States, seasonal influenza outbreaks occur every winter; however, the timing and severity of these seasonal outbreaks vary considerably from year to year. Accurate forecast of key characteristics of influenza outbreaks would allow public health agencies to better prepare for and respond to epidemics and pandemics. To this end, there has been significant work in recent years to develop forecasts of seasonal influenza outbreaks[1–5]. Proposed forecast methods include a range of statistical and mechanistic models, and incorporate data sources including syndromic and viral surveillance data, meteorological information, and internet search queries[3]. A comparison of coordinated influenza forecasts for the 2013–2014 season showed substantial disagreement in predicted outbreak characteristics among forecast methods[1]. This disagreement presents a barrier to the use of forecasts in decision-making.

While some forecast methods may be consistently superior to others, the relative performance of individual forecast methods can also vary according to the specifics of the population, location, or outbreak of interest. For example, a forecast method developed for a densely populated urban area may be less accurate in a sparse rural setting. Alternatively, some forecast methods may be better suited for prediction of outbreaks that are smaller or larger than typically observed. The optimal forecast method may also depend on when in the season the forecast is being generated.

Previous comparisons of subsets of the individual forecast methods used in this study have indeed revealed differences in forecast performance. Yang et al. [5] compared the performance of six filtering methods coupled with a mathematic model of disease transmission in retrospective forecasting of influenza epidemics. The relative performance of the filter methods varied by the timing of the forecast, the location being forecast, and the number of observed peaks in the outbreak. In retrospective forecasts of dengue fever, the relative accuracy of a model-filter forecasting system and two statistical methods varied by forecast timing, forecast target of interest (i.e. timing of outbreak peak, maximum incidence and total incidence), and the similarity between the outbreak being forecast and previously observed outbreaks[6].

In weather and climate forecasting, discordant forecasts from competing models are combined into superensemble forecasts in order to offset the biases of each individual model. The resulting superensemble forecasts are more accurate than forecasts from any one model form [7–9]. Recent work has shown this superensemble approach to be effective in improving the accuracy of forecasts of dengue outbreaks[6] and influenza [10, 11].

Here, we compare the accuracy of a suite of 21 competing forecast methods, as well as a superensemble forecast, in retrospective forecasts of influenza epidemics (See Methods and S1 Appendix). We group the 21 individual forecasts into three categories: ensemble filter systems; particle filter systems; and a statistical model. The ensemble-based filter systems include three types of filters: Ensemble Kalman Filter (EKF), Ensemble adjustment Kalman Filter, (EAKF) and Rank Histogram Filter (RHF). Two particle filters are used: a basic particle filter (PF) and a particle Markov Chain Monte Carlo method (pMCMC). The five filter methods are each coupled with four standard compartmental models of disease transmission: SIR, SEIR, SIRS and SEIRS (see Methods). For the final individual forecast method, we use a statistical model called Bayesian Weighted Outbreaks (BWO).

We find that the superensemble forecasts are more accurate than any individual forecast system, and that this advantage increases when superensemble weights are stratified according to the characteristics of the forecast, or by geographic location.

## Results

### Comparison of individual forecasts

For the ensemble and particle filter systems, observations of influenza incidence from the beginning of the season to the week of forecast initiation are used to optimize each of the 4 mathematical models. The optimized model is then run to the end of the season to generate a weekly forecast of influenza incidence, as measured by ILI+, an estimate of influenza positive patients per 100 patient visits to outpatient health care providers (see Methods). For the BWO, only the most recent 8 weeks are used for training. A forecast is then generated as a weighted average of historical ILI+ epidemic trajectories (see Methods).

Thus, each individual forecast method produces a weekly estimate of ILI+ from the time of forecast through the end of the influenza season. These trajectories were used to calculate key characteristics of each outbreak: the peak incidence, peak timing, and total incidence. Forecast skill was assessed based on the mean absolute error (MAE) in predictions of each of these metrics.

**Fig 1:**
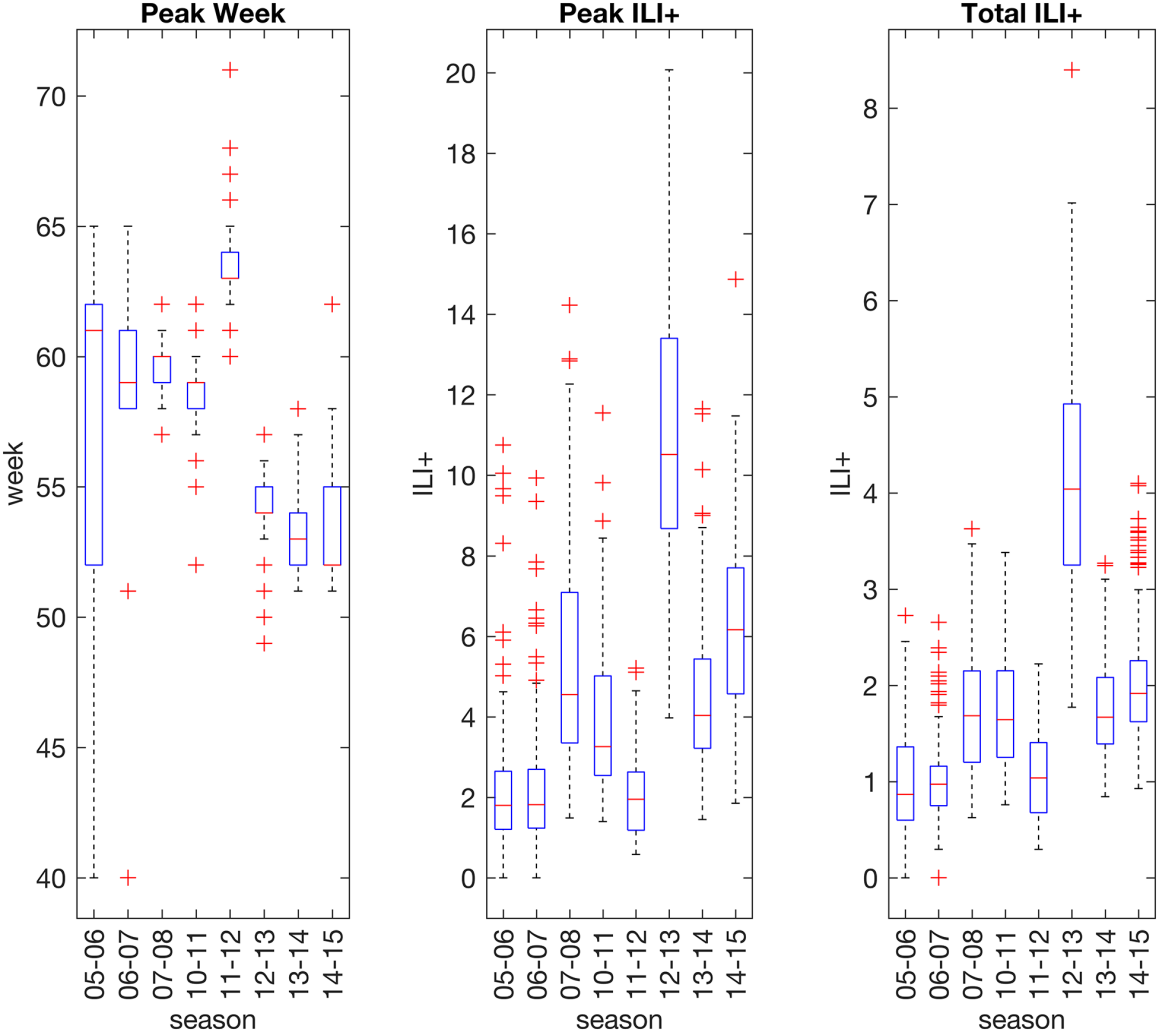
Distributions of observed outbreak peak timing, peak ILI+ and total ILI+ by season. Boxplots show the range of metric values observed each season across 97 cities and 48 states. ILI+ measures the number of influenza positive patients per 100 visits to outpatient visits to health care providers.

**Fig 2:**
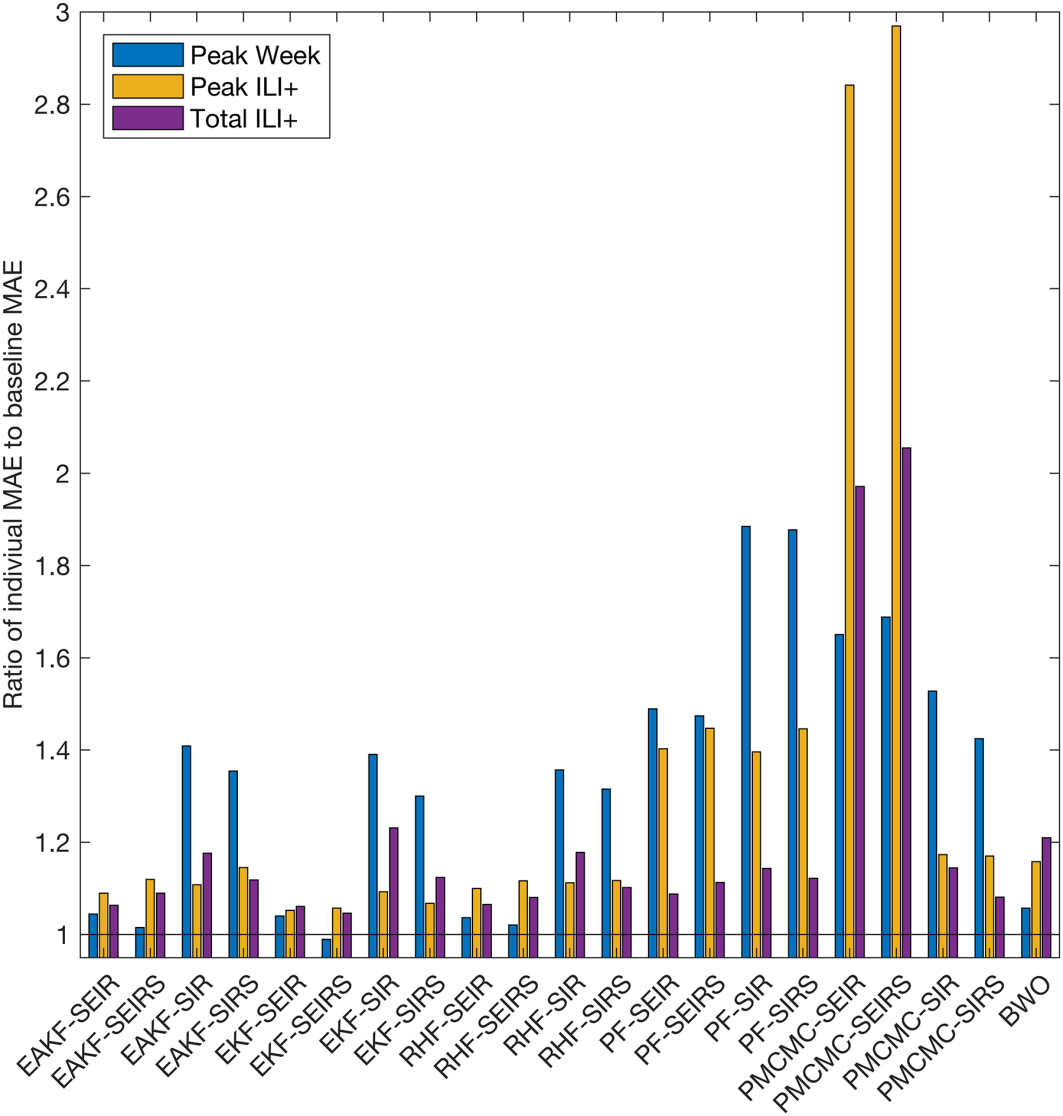
Ratio of individual forecast MAE to baseline level MAE. The baseline level MAE for each metric is the corresponding score of the baseline superensemble forecast (listed in Table 1). The three colors show the three target metrics. Note the abbreviated y-axis; the black line indicates a ratio of 1.

Distributions of seasonal influenza outbreak peak timing, peak ILI+ and total ILI+ across 48 states and 97 cities are shown in Fig 1. The overall error of each of the 21 individual forecast methods (see Methods for names and descriptions of each forecast system) used to predict these observed outbreak characteristics is shown in Fig 2, relative to the MAE of the Baseline superensemble as a reference value (discussed below in Superensemble results). Forecast MAE ranged from 1.8 weeks to 3.5 weeks for peak timing. Individual forecast MAEs for peak incidence ranged from 1.2 to 3.5 ILI+, and from 0.5 to 1.0 ILI+ for total incidence. Among individual forecast methods, the lowest MAEs for peak timing and total incidence were generated by the dynamical models that included an exposed compartment (i.e. the SEIR and SEIRS structures) coupled with ensemble filters. However, the advantage of the exposed compartment was less clear in forecasts of peak incidence. Rather, the EKF model-filter systems produced the most accurate forecasts of peak incidence. Ensemble filter methods and BWO consistently outperformed particle filter methods for peak week and peak incidence; for total incidence, several particle-filter forecast systems performed comparably to ensemble methods. The pMCMC-SEIR and pMCMC-SEIRS forecast systems had exceptionally large errors for peak and total incidence.

**Fig 3:**
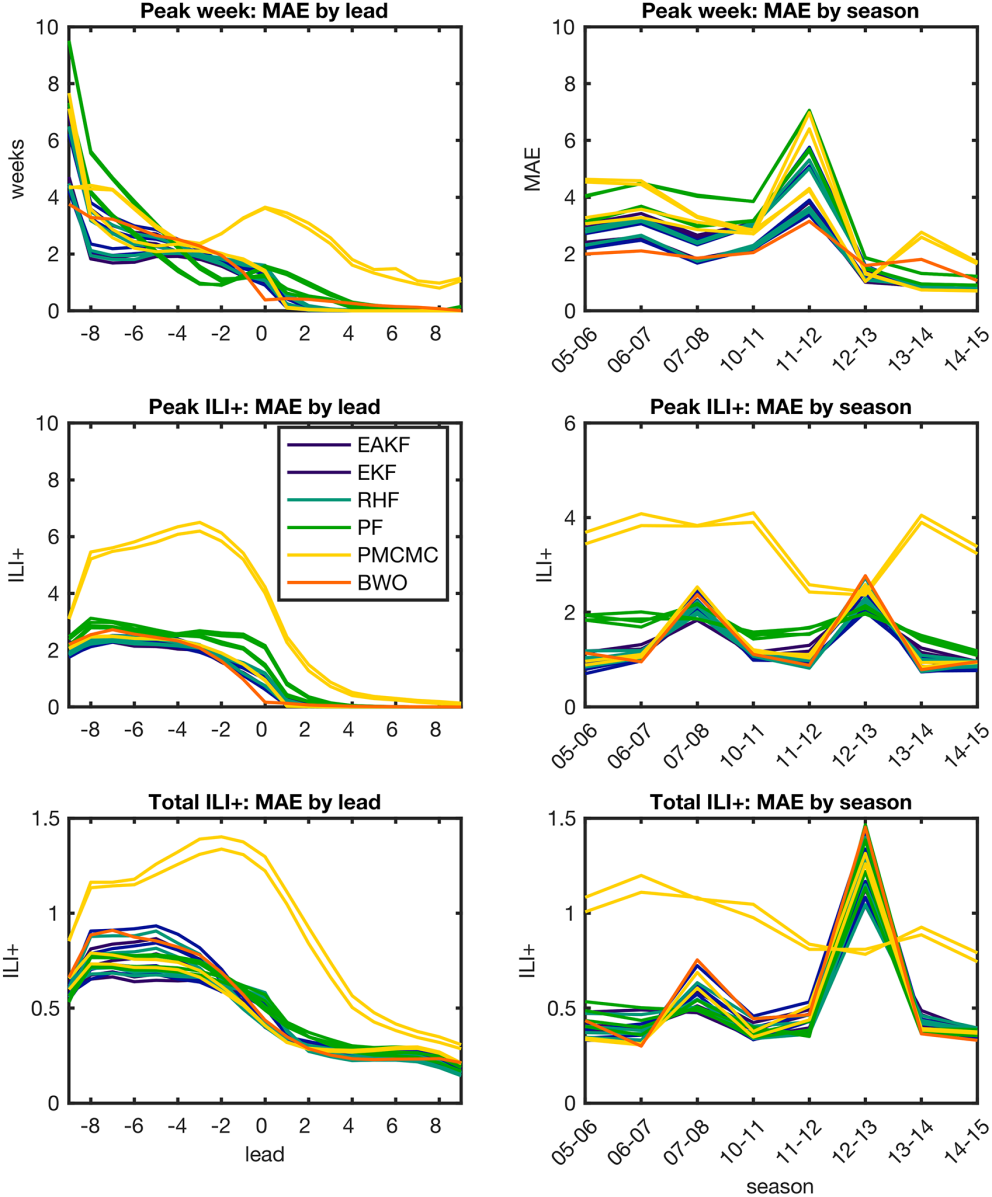
Forecast MAE grouped by forecast lead (left column) and season (right column). Line plots show MAE for forecast peak week (top row), peak ILI+ (middle row) and total ILI+ (bottom row), averaged over all locations. Each line shows the MAE of an individual forecast method. For the model-filter systems, colors indicate groupings by filter type.

Fig 3 shows the MAE of each individual forecast method by forecast lead time and influenza season. Lead time refers to timing of the forecast relative to the outbreak peak, in weeks, with negative values indicating forecasts made prior to the peak and positive values for forecasts made after the peak. There were clear differences in relative forecast accuracy when discriminating by these factors. For example, the ensemble filters and BWO generally outperformed the particle filters in forecasts of peak ILI+ and total ILI+, with the exception of 2007–2008 and 2012–2013, two years with relatively large outbreaks. The particle filters produced the most accurate forecasts of peak week 1 to 5 weeks before the peak, but were among the worst performers following the peak. Inaccurate predictions of outbreak peak are possible even after the true peak has passed, as the models may predict a continued increase in incidence resulting in a later peak. The pMCMC-SEIR and pMCMC-SEIRS systems were especially prone to this type of error. Unlike the other filter methods, the pMCMC requires the same set of parameters be used to fit the entire observed time series [5]. As a result, the filter is less able to adapt to shifts in outbreak dynamics during an influenza season. This can result in poor forecasts, particularly for multi-peak outbreaks.

### Superensemble results

**Fig 4:**
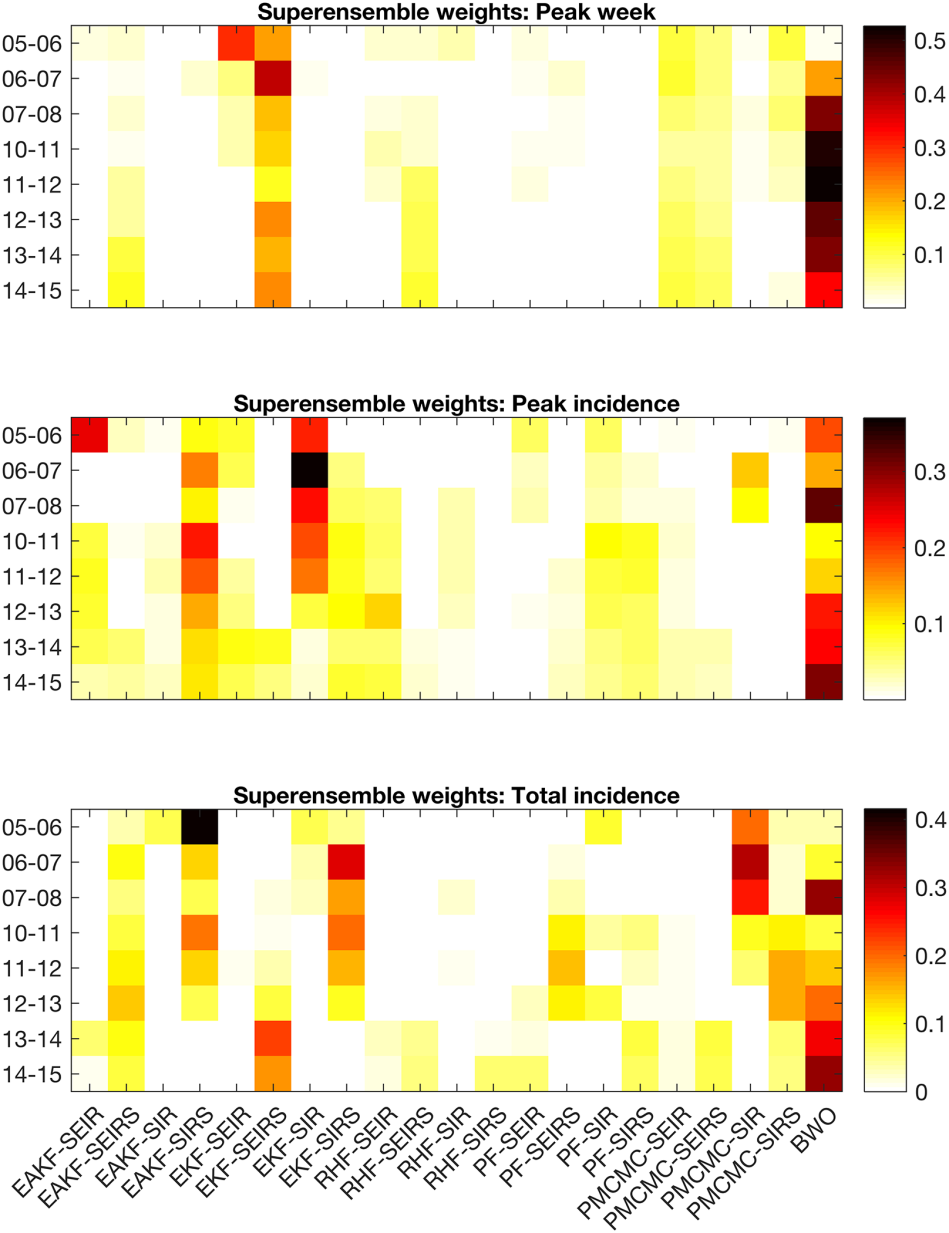
Contribution of each individual forecast to the baseline superensemble forecast for peak week (top), peak incidence (middle), and total incidence (bottom) by year. The colors indicate the weight assigned to an individual forecast system specified by the horizontal axes and the influenza season specified by the vertical axes. For example, the upper-right square in each subplot shows the weight assigned to the EAKF-SEIR forecast system for the 2005–2006 influenza season.

For the initial baseline set of superensemble forecasts, a single set of weights for each target metric was applied to the competing individual forecasts each season (Fig 4). Each season, a new set of superensemble weights were computed using training data from all previous years. As such, we expected increased stability in the weights as more years were used to generate the weights. For all target metrics, the BWO forecast was typically assigned large weight, ranging from .10 to .53 for peak week, .10 to .32 for peak ILI+, and .03 to .33 for total incidence. However, note that the BWO approach is the most dissimilar among the forecast approaches. If a subgroup of forecasts predicts similar outcomes, the superensemble weights, which sum to one, are expected to be split among that subgroup. The contribution of the individual model-filter forecasts, which share filter methods and model structures, may thus be diluted among similar forecasts. This circumstance may explain the heavy weighting of the BWO forecast, despite its larger than average MAEs among individual forecasts. The twelve ensemble filter systems contributed a total weight of .29 to .68 for peak week, .49 to .68 for peak incidence, and .36 to .69 for total incidence. The eight particle filter systems contributed a total weight of .14 to .31 for peak week, .14 to .21 for peak incidence., and .26 to .43 for total incidence.

The MAEs of superensemble forecasts are shown in Table 1. On average, the baseline superensemble forecasts were more accurate at predicting the timing of the outbreak peak than any of the individual forecasts, with the exception of the EKF-SEIRS (Fig 2). Baseline superensemble forecasts of peak and total incidence had smaller MAE than all individual forecasts.

**Table 1:** Mean absolute error for superensemble forecasts. Forecast error averaged over all seasons, forecast weeks, and locations.

Weighting Scheme	MAE		
	Peak Week	Peak ILI+	Total ILI+
Baseline	1.85	1.19	0.47
Week	1.72	1.18	0.47
HHS Region	1.86	1.15	0.45
Forecast lead	1.74	1.19	0.46
Actual lead	1.39	1.13	0.46
Population density	1.87	1.18	0.47
Population Size	1.85	1.18	0.47

**Fig 5:**
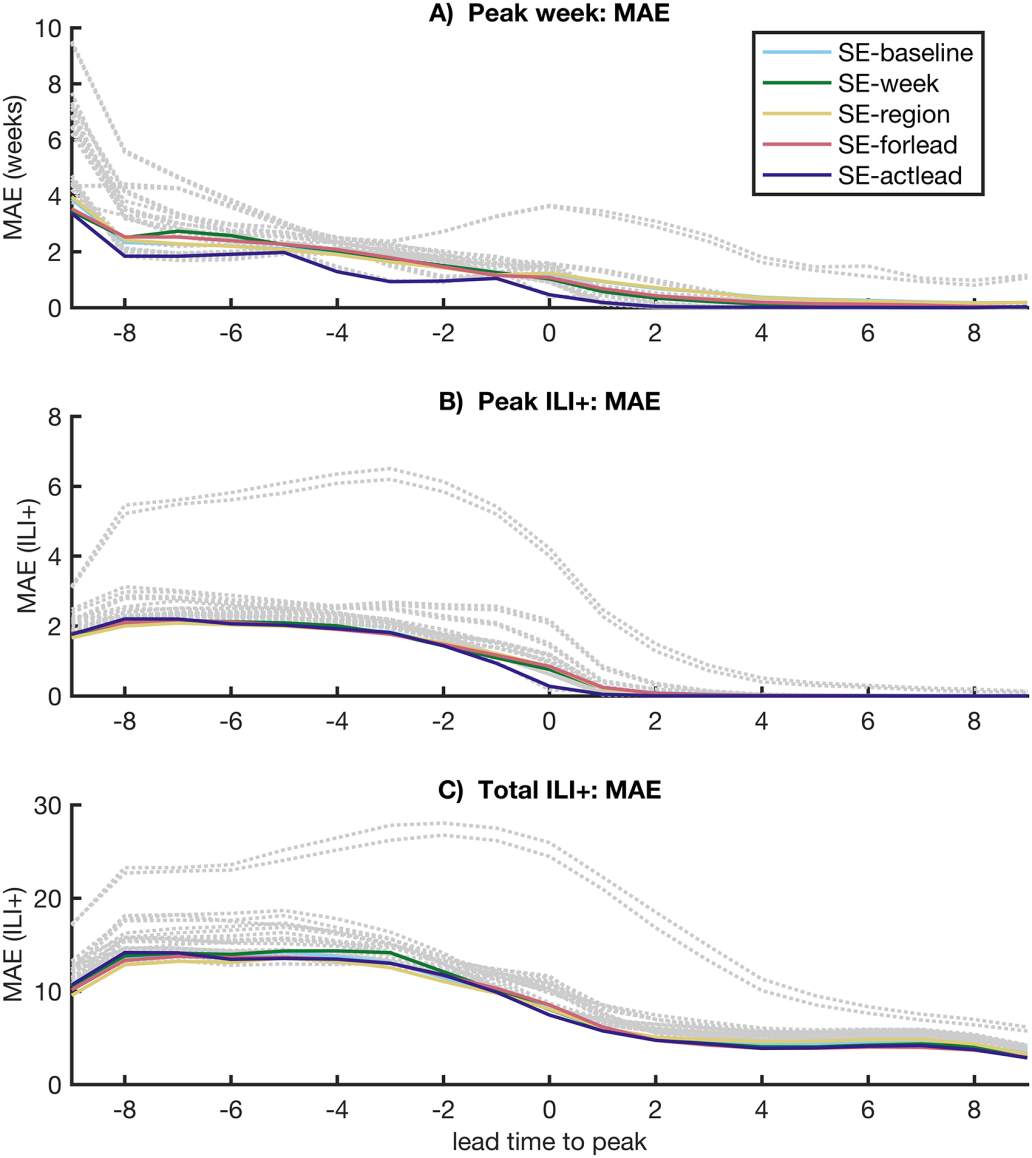
Performance of superensemble forecasts compared to individual forecasts stratified by lead time. Each line shows the results of one forecast, with grey dotted lines representing the 21 individual forecasts and colored lines representing superensemble forecasts. SE-baseline refers to the baseline superensemble forecast, while SE-week, SE-region, SE-forlead and SE-actlead refer to superensemble forecasts with weights stratified by forecast week, HHS region, lead relative to predicted peak, and lead relative to observed peak, respectively.

We then stratified the weights by geographical region, forecast week, lead time relative to predicted peak, lead time relative to observed outbreak peak, population size and population density. These variables were pre-specified on the basis of previous work indicating that they may influence the accuracy of individual forecast methods (for example [5, 6]). The MAEs of the stratified superensemble forecasts are shown in Table 1. For both peak timing and peak incidence, stratifying by lead time relative to observed peak led to the greatest improvements in forecast performance, decreasing MAE by 0.4 weeks for peak timing and 0.06 ILI+ for peak incidence compared to the baseline superensemble forecast. On average, this forecast outperformed individual forecasts of peak timing and peak incidence at all times during the course of the outbreak (Fig 5). In contrast, several individual forecasts were more accurate in forecasting peak timing than the baseline superensemble early in the season, while others outperformed the baseline superensemble late in the season (Fig 5).

Averaged within influenza seasons, the superensemble forecasts generally outperformed individual forecasts (S1 Fig) The most notable single seasonal exception was the set of forecasts of total incidence in 2012–2013, the year with unusually high influenza incidence.

Stratifying superensemble weights by forecast week or lead time relative to predicted peak, which can serve as real-time proxies for actual lead time, led to decreases in MAE for peak timing, but had only a small effect on forecasts of peak and total incidence. Meanwhile, stratifying by geographical region and population density led to small decreases in MAE for peak incidence, but degraded forecast accuracy for peak timing (Table 1, Fig 5). Stratifying weights by geographical region led to the lowest MAE for total incidence, but the improvement over the baseline forecast was small (0.02 ILI+).

We further assessed the accuracy of superensemble forecast credible intervals by determining the fraction of observations falling within the credible intervals specified by each forecast. In a well-calibrated forecast, we would expect this fraction to correspond to the value of the credible intervals; for example, 95% of observed outcomes should fall within the 95% credible intervals of a forecast method. Overall, the forecasts were well-calibrated. The calibration varied between the three target metrics, as well as between choices of stratification variable for superensemble weighting (S2 and S3 Figs) Forecasts of peak week and peak ILI+ were well calibrated at 90% and 95% credible intervals but somewhat overdispersed at lower confidence intervals. Forecast coverage for total ILI+ was well calibrated for 50%, but underdispersed at 95% and 99% credible intervals.

### Forecast rankings

**Fig 6:**
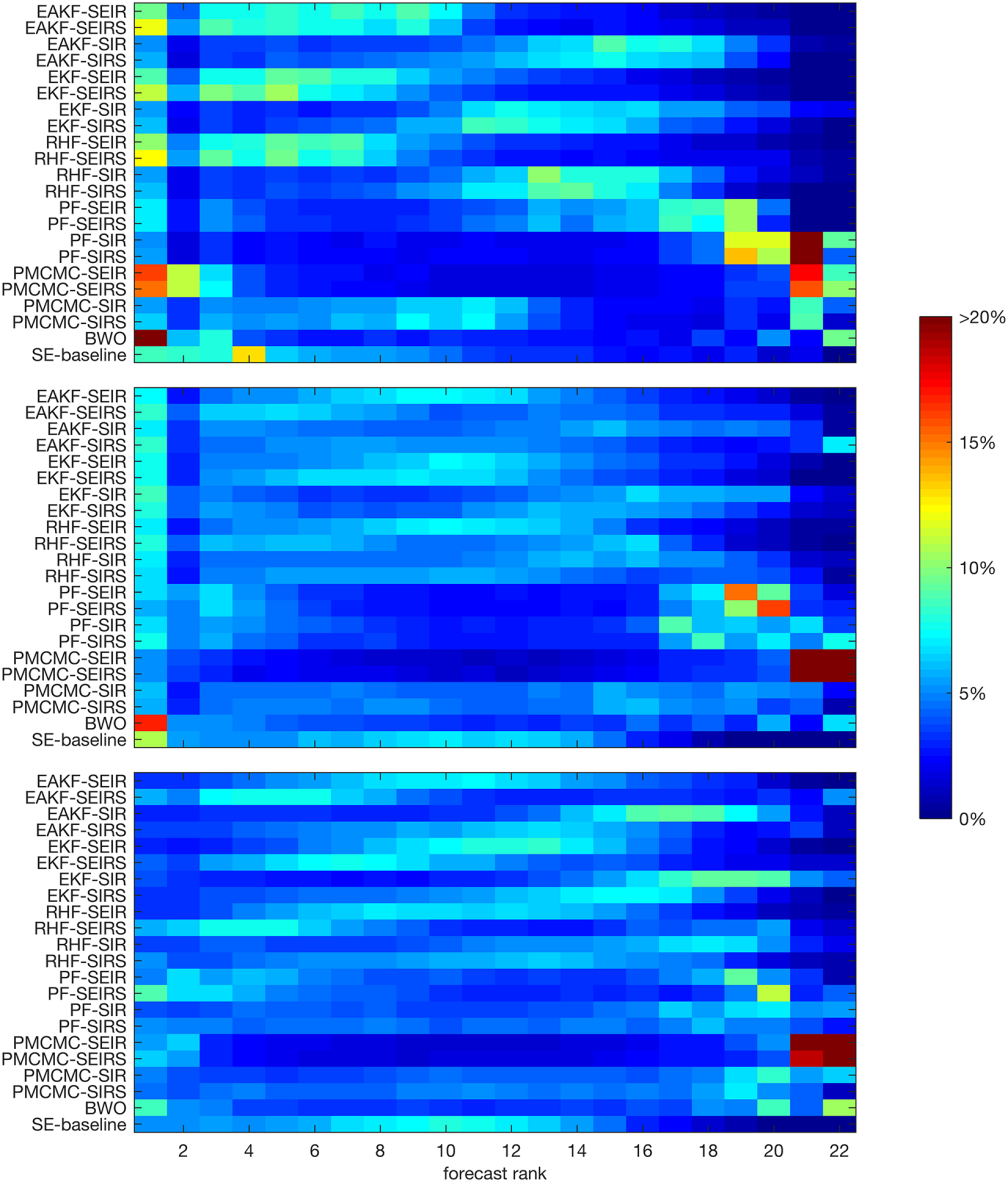
Heat map of forecast rankings. The colors indicate the frequency of each forecast ranking, with rank 1 assigned to the most accurate forecast and rank 22 assigned to the least accurate forecast. More optimal forecasts have a higher frequency of top rankings (warm colors on the left) and a lower frequency of bottom rankings (cold colors on the right). The upper image shows peak week, the middle shows peak incidence, and the bottom shows total incidence. This analysis includes all forecasts of total season incidence (n = 22640), and all forecasts made at or prior to the observed outbreak peak for peak week and peak incidence (n = 15187). Forecasts made after the peak had been observed were excluded from the ranking, as were forecasts where all 22 forecasts predicted the same outcome.

In addition to comparing forecast errors averaged over many forecasts, we also compiled a ranking of individual forecast outcomes. For each forecast, we ranked the 21 point-estimates from the individual forecasts and the resulting baseline superensemble forecast from 1 (lowest absolute error) through 22 (highest absolute error) and summed the frequency of each ranking (Fig 6). For peak week and peak incidence, we restrain the analysis to forecasts made prior to the observed forecast peak, as most forecasts report the true peak values after the peak has been observed, resulting in equal ranking.

The superensemble gave the most accurate forecast 8.6% of the time for peak timing, which was more frequently than 12 of the individual forecast methods, but less frequently than BWO and most models-filter combinations using SEIR and SEIRS models. While the BWO, pMCMC-SEIR and pMCMC-SEIRS forecasts of peak timing had the most frequent first place rankings, they also produced many predictions that received the lowest rankings. In contrast, the superensemble forecast, as well as the SEIR and SEIRS models coupled with EAKF, EKF, and RHF ensemble filter methods, had few low rankings. When superensemble weights were stratified by lead time of forecast relative to the observed outbreak peak, the resulting forecast rankings dramatically improved, with 54.3% of superensemble forecasts receiving a rank of 1 through 4, surpassing all individual forecast methods (S4 Fig).

In forecasts of peak incidence, the baseline superensemble forecast gave the best prediction 10.8% of the time, which was more frequent than all individual forecasts except BWO, which was the highest ranked method for 17.1% of predictions. The superensemble was among the 4 worst forecasts less than 0.5% of the time, compared to 17.7% of BWO forecasts (Fig 6). The proportion of high to low ranking forecasts increases when superensemble weights are stratified by lead time of forecast (S4 Fig). These results indicate that the superensemble provides a consistent advantage in forecasts of peak incidence, both in aggregate, as well as for any given prediction.

For forecasts of total incidence, the baseline superensemble forecast had an average number of first place forecasts (5%), and was most often ranked between 7 and 13. However, as with the other two metrics, the superensemble had far fewer low rankings than any individual method. Among individual ensemble models, those using the SEIRS structure were ranked highest. BWO, pMCMC-SEIR, pMCMC-SEIRS, PF-SEIR and PF-SEIRS had frequent rankings in both the top 4 and the bottom 4.

## Discussion

Disagreement between competing forecasts of infectious disease outbreaks presents an obstacle to the interpretation and utilization of such forecasts. Here, we have presented a method for reconciling the disagreement among forecasts, while simultaneously improving overall forecast accuracy. We have shown that overall forecast accuracy for the timing and magnitude of peak influenza transmission is improved by combining individual forecasts into weighted-average superensemble forecasts. These superensemble forecasts were, on average, more accurate than any individual forecast method. In particular, the superensemble was less prone to producing a poor forecast. The advantage of the superensemble approach increases in circumstances where the relative accuracy of individual forecasts varies according to characteristics of the outbreak, or the location being forecast.

The 21 individual forecast methods compared in this study varied in their performance, as well as their reliability. The SEIRS dynamical model coupled with the EKF, EAKF and RHF ensemble filter methods were consistently among the better individual forecasts. Other forecast methods, namely pMCMC-SEIRS and pMCMC-SEIR, and to a lesser extent, BWO, performed inconsistently in that predictions were either among the best or the worst of the competing forecasts. This type of inconsistent performance presents a challenge to the superensemble approach, as the good forecasts can cause the forecast to receive a relatively high weighting in the superensemble; however, by identifying the circumstances that lead to differences in relative forecast performance, adaptive weighting can then be employed to variably weight an individual forecast method highly when it is prone to perform well and discount it in other circumstances. Here we found that the performance of individual forecast methods varied according to geographic location, influenza season, and the timing of the forecast.

We found that the timing of the forecast with respect to the outbreak peak was an important factor in determining relative forecast accuracy; consequently, stratifying superensemble weights by the actual lead time of the forecasts led to improvements in superensemble forecast accuracy. This improvement outweighed the gains made by simply eliminating the two most inconsistent forecast methods (pMCMC-SEIR and pMCMC-SEIRS). While the idealized process of weighting individual forecasts by actual forecast lead is not possible in real time, stratifying weights by forecast-predicted lead or simply calendar week, which can serve as real-time proxies for actual forecast lead, proved beneficial in improving forecast accuracy.

Stratifying superensemble weights by HHS region improved forecasts of peak and total incidence. This benefit may be related to regional differences in baseline and seasonal levels of influenza activity, or could be reflective of differences in the progression of influenza among regions. These findings provide a robust methodology for generating superensemble forecasts of influenza and other infectious diseases; however, the superensemble weights and the optimal stratification partitioning must be continually reevaluated and updated as new years of data become available, or as the geographical scale or resolution are altered.

While this study focused on the forecast of point estimate outcomes, the superensemble approach can also be used to produce probability distribution functions of target metrics. For example, the superensemble forecasts presented here were associated with reasonable credible intervals, which were influenced by the choice of stratification variable (S3 Fig). The calibration of individual and superensemble probabilistic forecasts remains an area of ongoing research.

The algorithm used to create the superensemble is flexible, and can combine any number and any type of individual forecast method, provided retrospective forecasts are available for superensemble training. These findings can thus be applied operationally to competing forecasts of infectious disease in order to improve forecast accuracy, and to present a streamlined prediction to public health decision-makers.

## Methods

### Observations of influenza activity

Regional influenza activity is monitored by the U.S. Centers for Disease Control and Prevention (CDC) through the U.S. Outpatient Influenza- like Illness Surveillance Network (ILINet). The CDC provides weekly near real-time estimates of regional influenza-like illness (ILI), defined as the number of patients with flu-like symptoms (fever with sore throat and/or with cough) divided by the total number of patient visits at ILINet outpatient healthcare facilities in order to account for temporal and spatial variability in patient volume and reporting rates of health care providers [1]. At the city and state level, ILI was estimated by Google Flu Trends (GFT), which used a statistical model relating weekly CDC ILI data to Google internet search queries[12]. GFT estimates are available for up to 115 cities and 50 states, from 2003 until the program was discontinued in 2015.

ILI is not specific to influenza, as it encompasses a range of respiratory infections. The World Health Organization and National Respiratory and Enteric Virus Surveillance System (NREVSS) provide weekly reports of the proportion of laboratory-confirmed positive tests for influenza virus. A more specific estimate of influenza activity can be obtained by multiplying ILI with corresponding weekly regional viral isolation information, resulting in a measure we refer to as ILI+, defined as the number of influenza positive patients per 100 patient visits. As in our previous studies, we use city and state level GFT ILI estimates multiplied by regional NREVSS viral isolation rates to obtain ILI+, as the metric of observed influenza incidence [5].

### Forecast targets

We produced weekly forecasts of three target metrics for each influenza outbreak: the highest observed weekly ILI+ (peak incidence); the week during which peak ILI+ occurred (peak week); and the total ILI+ over the influenza season, which we define as a 20-week period beginning on the 45^th^ calendar week of the year (total incidence).

### Forecast methods

Weekly forecasts of influenza outbreak trajectories, outbreak peak ILI+, total ILI+ and outbreak peak timing were produced using 21 different forecast methods. Of the 21 methods, 20 are variations of a mathematical model of disease transmission coupled with a data assimilation, or filtering, method, and 1 is a statistical model based on historically observed outbreaks. We generated retrospective forecasts for 95 cities and the 48 contiguous United States with available records during the 2005–2006 through 2014–2015 influenza seasons, excluding the pandemic seasons of 2008–2009 and 2009–2010. Pandemic seasons were excluded because the individual forecast systems used in this study were designed specifically for seasonal outbreaks. While the model-filter forecasts could be adapted to forecast pandemics or irregularly timed outbreaks (for example [13–15]), the Bayesian Weighted Outbreaks method in particular is not appropriate for forecasting pandemics as it relies on the assumption that the outbreak in progress will be similar in timing and magnitude to previously observed outbreaks.

#### Mathematical models of disease transmission

Our previous studies using model-filter forecasts of influenza feature a humidity-forced Susceptible-Infectious-Recovered-Susceptible (SIRS) model to simulate influenza transmission [2, 5, 16]. This model assumes a perfectly-mixed population and is described by the following equations.

$$\frac{dS}{dt}\;=\;\frac{N-S-I}{L}-\;\frac{\beta (t)IS}{N}-\alpha$$ (1)

$$\frac{dI}{dt}\;=\;\frac{\beta (t)IS}{N}-\frac{I}{D}+\alpha$$ (2)

where *N* is the population size, *S* and *I* are, respectively, the number of susceptible and infectious individuals in the population, and *N-S-I* is the number of recovered (immune) individuals. The model parameters are: *D*, the mean duration of infection; *β(t)*, the transmission rate at time *t*, *L*, the mean duration of immunity; and α, the rate of travel-related imported influenza into the model domain.

The transmission rate *β(t)* is related to the basic reproductive number, *β(t)* = *R*_*0*_ *(t)/D*. *R*_*0*_*(t)* is modulated daily based on the empirical relationship between absolute humidity and viral transmission[17]:

$$R_{0}(t)\;=\;R_{0min}+(R_{0max}-R_{0min})e^{-180q(t)}$$ (3)

where *R*_*0min*_ and *R*_*0max*_ are the minimum and maximum daily basic reproductive numbers, respectively, and *q(t)* is time-varying daily average specific humidity.

In addition to the SIRS model, we consider three alternate model structures: Susceptible-Infectious-Recovered (SIR); Susceptible-Exposed-Infectious-Recovered (SEIR); and Susceptible-Exposed-Infectious-Recovered-Susceptible (SEIRS). The Exposed compartment in the SEIR and SEIRS models represent a latent period of infection. The equations describing these additional model structures are provided in S1 Appendix.

#### Filter methods

The mathematical models described above are coupled with filter methods for the weekly assimilation of ILI+ observations and optimization of the model state variables and parameters. These filters use Bayesian inference to calculate the posterior conditional distribution of parameters and state variables given observed ILI+, based on the prior distribution of the model ensemble and the conditional distribution of observed ILI+ given model parameters and state variables.

Five different data assimilation methods were used with each of the four model structures. These consist of three ensemble filter methods—the ensemble Kalman filter (EKF)[18], the ensemble adjustment Kalman filter (EAKF)[19] and the rank histogram filter (RHF)[20], and two particle filter methods—a basic particle filter (PF) with resampling and regularization[21] and the particle Markov chain Monte Carlo (pMCMC) method[22]. A description of each filter method is provided in S1 Appendix. For full filter details, we refer readers to the original texts referenced above.

The combined model-filter system recursively updates the model state variable and parameter estimates with each new weekly ILI+ observation. Through this process, these estimates of the observed and unobserved variables and parameters values are nudged closer to their true values. Following the assimilation of the most recent weekly ILI+ observation, the optimized set of model simulations is propagated forward in free simulation through the remainder of the season, producing a forecast of future ILI+ observations, from which we calculate the predicted timing and magnitude of peak influenza incidence, and total incidence.

#### Bayesian weighted outbreaks

The final forecast method, which we call Bayesian weighted outbreaks (BWO), is a statistical method that uses Bayesian model averaging to describe the trajectory of ILI+ for the outbreak of interest as a weighted average of outbreak trajectories from prior seasons. This method has been used in weather forecasting[23], and has previously been adapted to forecast dengue outbreaks[6]. The weights assigned to candidate trajectories are determined based on the likelihood of the ILI+ values observed for the outbreak in progress given ILI+ during the same time period (weeks *t*-7 through *t*) in the candidate trajectories (see [6] for further details). The forecast trajectory of ILI+ obtained by taking the weighted average is used to predict peak timing, peak incidence and total incidence in the outbreak of interest.

The pool of candidate trajectories used in the BWO retrospective forecasts included ILI+ observations from all 145 locations (48 states and 97 cities) available for the seasons prior to the season being forecast (e.g. retrospective forecasts of the 2005–2006 season considered ILI+ trajectories from 2003–2004 and 2004–2005—seasons 1 and 2), excluding the pandemic years of 2008–2009 and 2009–2010. More generally, retrospective BWO forecasts of influenza season *N* use candidate trajectories from seasons 1 through N-1.

### Superensemble model averaging algorithm

Superensemble forecasts were created for the 2005–2006 through 2014–2015 influenza seasons (excluding the pandemic seasons 2008–2009 and 2009–2010) by taking the weighted average of the 21 individual weekly forecasts for each location. The superensemble weights, which dictate the contribution of each individual forecast to the superensemble, are determined using maximum likelihood estimation of the conditional probability distribution function (PDF) over a selected number of training forecasts:

$$p(y'|{f'}_{1,m},\ldots ,{f'}_{21,m})\;=\;\sum_{k\;=\;1}^{21}w_{k,m}g_{k}(y'|{f'}_{k,m})$$ (4)

where the left hand side of the equation is the probability distribution of the superensemble forecast, and the right hand side is the weighted sum of the 21 individual forecast distributions, *g*_*k*_*(y’|f’*_*k*,*m*_*)*. More formally, *y’* is the true value of the training outbreak metric *m* (peak timing, peak incidence or total incidence), *w*_*k*,*m*_ is the probability that individual forecast method *k* is the most accurate method, and *g*_*k*_*(y’|f’*_*k*,*m*_*)* is the PDF of *y’*, conditional on training forecast *f’*_*k*,*m*_, given that *f’*_*k*,*m*_ is the most accurate forecast of *y’*_*m*_. This conditional PDF is assumed to be normal with mean *f’*_*k*,*m*_ and standard deviation σ. For simplicity, σ is assumed equal for all individual forecasts, and is determined through the maximum likelihood estimation of Eq 4 to obtain *w*_*k*,*m*_, which serve as the superensemble weights (see Raftery et al.[23] for full details).

Superensemble weights for season *N* are trained using individual forecasts from 2003–2004 through season *N-1*. The set of BWO training forecasts, *f’*_*BWO*,*m*_, are produced using a leave-one-out approach for training seasons 1 through N-1. That is, training forecasts for each season from 1 through N-1 were constructed using trajectories using all other seasons between 1 and N-1. The number and diversity of candidate trajectories increases over time, as each subsequent year adds 145 additional ILI+ trajectories to the pool. The model-filter forecasts do not use historical observations, and thus do not require a leave-one-out approach for training forecasts.

The superensemble weights *w*_*k*,*m*_ are then applied to the point estimates of the target metric from the 21 individual forecasts, *f*_*k*_, for the time *t*, location *l*, and metric *m* of interest to obtain the superensemble forecast, SE:

$$SE_{m}(t,l)=\overset{21}{\underset{k=1}{\sum }}w_{k,m}f_{k,m}(t,l)$$ (5)

The probability distribution function of the superensemble forecast obtained from Eq 4 is used to determine credible intervals around each forecast (for example, S2 Fig). The width of the credible intervals is a function of both the spread of the point estimates from the 21 individual forecast methods, as well as the estimated variance of each individual forecast over the training period (*σ*^2^) [23].

### Superensemble weighting schemes

The baseline superensemble forecasts were made by applying a single set of superensemble weights across all locations and times within an influenza season. However, based on previous analyses of individual forecast system performance (e.g. [2, 5, 6]), we hypothesized that superensemble performance would improve if superensemble weights were stratified by the following variables: calendar week of forecast, lead time relative to forecast peak (weeks between the week of forecast initiation and the predicted peak), geographic region, population size, and population density.

A final set of forecasts was produced by stratifying superensemble weights by lead time relative to the actual peak (weeks between the week of forecast initiation and the week of the true peak). While this weighting scheme could not be implemented in an operational real-time forecast, it is useful to know how the superensemble would perform under this idealized condition, as this may represent an upper bound to improvements that can be achieved using a weighting scheme based on forecast timing.

The method for stratifying superensemble weights consisted of dividing forecasts into bins according to the variable of interest. We then obtained weights for each bin by including only training forecasts falling into that bin in the algorithm described in Eq 4. In setting the bin sizes for each variable, our objective was to resolve potential differences in forecast performance. This objective was balanced by the need to include sufficient training forecasts in each bin to avoid over-fitting.

Forecasts stratified by lead time (actual or predicted) were grouped using the following bin edges, where negative values indicate weeks prior to the peak and positive numbers are weeks after the peak: [<-8, -8, -7, -6, -5, -4, -3, -2, -1, 0, 1, 2, 3, 4, 5–8, 9–12, >12]. We selected a fine resolution of 1 week around peak, with wider bins at either end, as fewer outbreaks have lead times in these categories. Actual lead time is simply the week of the forecast minus the week that peak is eventually observed. Forecast predicted lead time is calculated by taking the mean prediction of peak week from the 21 individual forecasts, and subtracting this mean value from the week of forecast.

Geographic regions were grouped according to the ten US Health and Human Services Regions, as these are the standard geographical groupings used by the CDC to describe influenza activity. Calendar week groupings were delineated by individual weeks. Population density and populations size were arbitrarily binned into quintiles for cities and terciles for states.

## Supporting information

S1 Appendix Additional details on forecast methods. (PDF)

S1 Fig Performance of superensemble forecasts compared to individual forecasts by influenza season. Each line shows the results of one forecast, with grey dotted lines representing the 21 individual forecasts and colored lines representing superensemble forecasts. SE-baseline refers to the baseline superensemble forecast, whereas SE-week, SE-region, SE-forlead and SE-actlead refer to superensemble forecasts with weights stratified by forecast week, HHS region, lead relative to predicted peak, and lead relative to observed peak, respectively.
(TIF)

S2 Fig Sample superensemble forecast with 95% credible intervals. The weekly SE-baseline and SE-week forecasts are shown for a sample outbreak. 95% credible intervals are indicated by the shaded areas.
(TIF)

S3 Fig Coverage of forecast credible intervals. The points on the graph show the percent of observations falling within the specified credible intervals of the superensemble forecasts.
(TIF)

S4 Fig Heat map of forecast rankings. Same as Fig 6 in main text of paper, but with superensemble weights stratified by lead time of forecast relative to observed outbreak peak.
(TIF)
