# Supplementary material for: Individual versus superensemble forecasts of seasonal influenza outbreaks in the United States
Source: PLoS Comput Biol. 2017 Nov 6;13(11):e1005801. doi: 10.1371/journal.pcbi.1005801 (PMC5690687; doi:10.1371/journal.pcbi.1005801)
Supplement: S1 Appendix — (PDF) [file pcbi.1005801.s001.pdf]

S1 Appendix. Additional details on forecast methods.

1 Model structures

SIR model structure:

$$\frac{dS}{dt} = -\frac{\beta(t)IS}{N} - \alpha \tag{S1}$$

$$\frac{dI}{dt} = \frac{\beta(t)IS}{N} + \alpha \tag{S2}$$

SEIR model structure:

$$\frac{dS}{dt} = -\frac{\beta(t)IS}{N} - \alpha \tag{S3}$$

$$\frac{dE}{dt} = \frac{\beta(t)IS}{N} - \frac{E}{Z} + \alpha \tag{S4}$$

$$\frac{dI}{dt} = \frac{E}{Z} \tag{S5}$$

where E is the number of exposed people, and Z is the mean latent period.

SEIRS model structure:

$$\frac{dS}{dt} = \frac{N - S - E - I}{L} - \frac{\beta(t)IS}{N} - \alpha \tag{S6}$$

$$\frac{dE}{dt} = \frac{\beta(t)IS}{N} - \frac{E}{Z} + \alpha \tag{S7}$$

$$\frac{dI}{dt} = \frac{E}{Z} - \frac{I}{D} \tag{S8}$$

We assume a fixed α of 0.1 infections per day, and use a population size of 100,000 people.

2 Filter methods

Each of the model-filter forecast systems described in the main text uses one of the four possible mathematical models of disease transmission (SIR, SEIR, SIRS or SEIRS), and one of five filter, or data assimilation, methods. The main features of the five filter methods are described here. For full descriptions of the filter algorithms and implementations, we refer readers to the original publications.

2.1 Ensemble filter methods

Ensemble filter methods use an ensemble of model simulations, in this study 300, with parameters and state variables randomly initialized and iteratively optimized following each weekly ILI+ observation in a prediction-update cycle. In the prediction step, state variables are propagated forward in time by the disease transmission model until the next ILI+ observation becomes available. In the update step, the filter algorithms adjust ensemble members in order to better match the observation. The updates applied to unobserved state variables are linear mappings from the update applied to the observed variable based on the prior ensemble covariance between the observed and unobserved variables.

The three ensemble filter methods differ in the calculation of the update of the observed variable (ILI+). In the ensemble Kalman filter (EKF)[1], the posterior of each model ensemble member is computed as the weighted average between the ensemble member and the ILI+ observation, with Gaussian random noise around the observation consistent with the observational error variance. The weights are determined according to the ratio of the overall ensemble prior variance to the observational error variance.

The ensemble adjustment Kalman filter (EAKF)[2] deterministically computes the update step such that the posterior ensemble mean and variance match the mean and variance predicted by Bayes theorem, assuming a Gaussian distribution.

While the EKF and the EAKF assume a Gaussian structure in prior and posterior ensemble distributions and observations, the rank histogram filter (RHF)[3] relaxes these assumptions and allows for non-Gaussian structures. Instead, an approximate continuous prior distribution is constructed using a rank histogram of ensemble prior values of ILI+.

This non-Gaussian prior is multiplied by the observational likelihood at each point and normalized, resulting in a continuous non-Gaussian posterior distribution.

A multiplicative inflation factor of 1.02 is added to the three ensemble filters to counter filter divergence. [2]

2.2 Particle filter methods

Particle filters represent state space with a set of particles, in this case 10,000. As with the ensemble filters, we couple the filter with a disease transmission model, which propagates the particles forward in time in a prediction step. The filter then assimilates the next ILI+ observation in an update step. The update step in the basic particle filter (PF)[4] weights particles according to their likelihood. Resampling and regularization improve the performance of the PF by expanding the range of parameter and state space, and decreasing redundancies.

The second particle filter is the particle Markov Chain Monte Carlo filter (pMCMC), specifically the particle marginal Metropolis-Hasting sampler described in Andrieu et al.[5], which combines Markov Chain Monte Carlo (MCMC) and sequential Monte Carlo (SMC) methods. While traditional SMC methods require sampling the entire state-parameter space of a model, the pMCMC simplifies the problem to sampling only parameter space. The pMCMC proposes a set of set of parameters, and then estimates state variables given the parameter proposal. The acceptance probability of the proposal is a function of the joint likelihood of the observed ILI+ data. Unlike the other four filters, in which parameters may be non-stationary in time, the pMCMC optimizes a fixed set of parameters over the entire observational time series.

References

1. Burgers G, Jan van Leeuwen P, Evensen G. Analysis scheme in the ensemble Kalman filter. Monthly weather review. 1998;126(6):1719-24.
2. Anderson JL. An ensemble adjustment Kalman filter for data assimilation. Monthly weather review. 2001;129(12):2884-903.
3. Anderson JL. A non-Gaussian ensemble filter update for data assimilation. Monthly Weather Review. 2010;138(11):4186-98.
4. Arulampalam MS, Maskell S, Gordon N, Clapp T. A tutorial on particle filters for online nonlinear/non-Gaussian Bayesian tracking. IEEE Transactions on signal processing. 2002;50(2):174-88.
5. Andrieu C, Doucet A, Holenstein R. Particle markov chain monte carlo methods. Journal of the Royal Statistical Society: Series B (Statistical Methodology). 2010;72(3):269-342.
