# Supplementary figures and images for: Individual versus superensemble forecasts of seasonal influenza outbreaks in the United States
Source: PLoS Comput Biol. 2017 Nov 6;13(11):e1005801. doi: 10.1371/journal.pcbi.1005801 (PMC5690687; doi:10.1371/journal.pcbi.1005801)

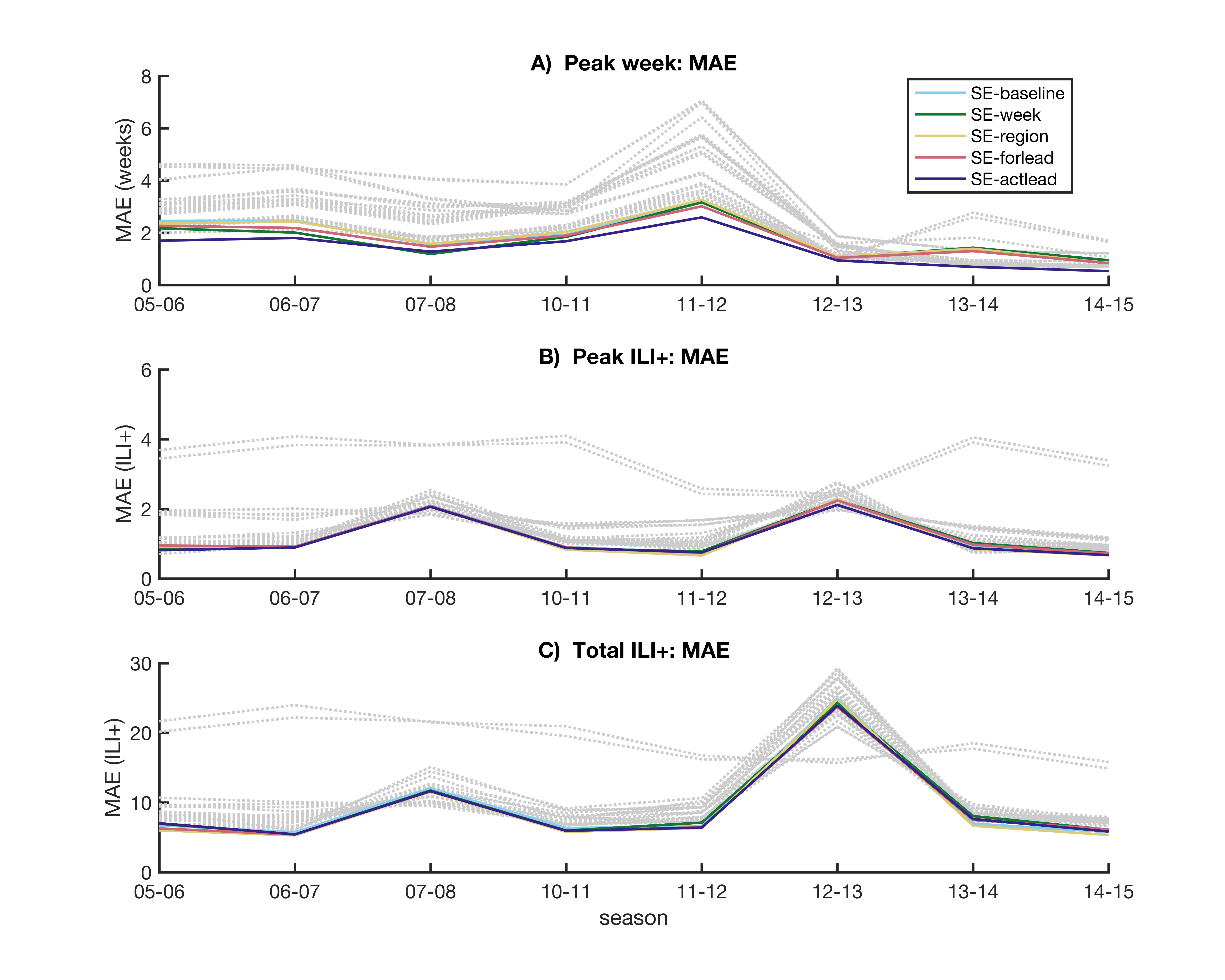

Supplement: S1 Fig — Each line shows the results of one forecast, with grey dotted lines representing the 21 individual forecasts and colored lines representing superensemble forecasts. SE-baseline refers to the baseline superensemble forecast, whereas SE-week, SE-region, SE-forlead and SE-actlead refer to superensemble forecasts with weights stratified by forecast week, HHS region, lead relative to predicted peak, and lead relative to observed peak, respectively. (TIF) [file pcbi.1005801.s002.tif]

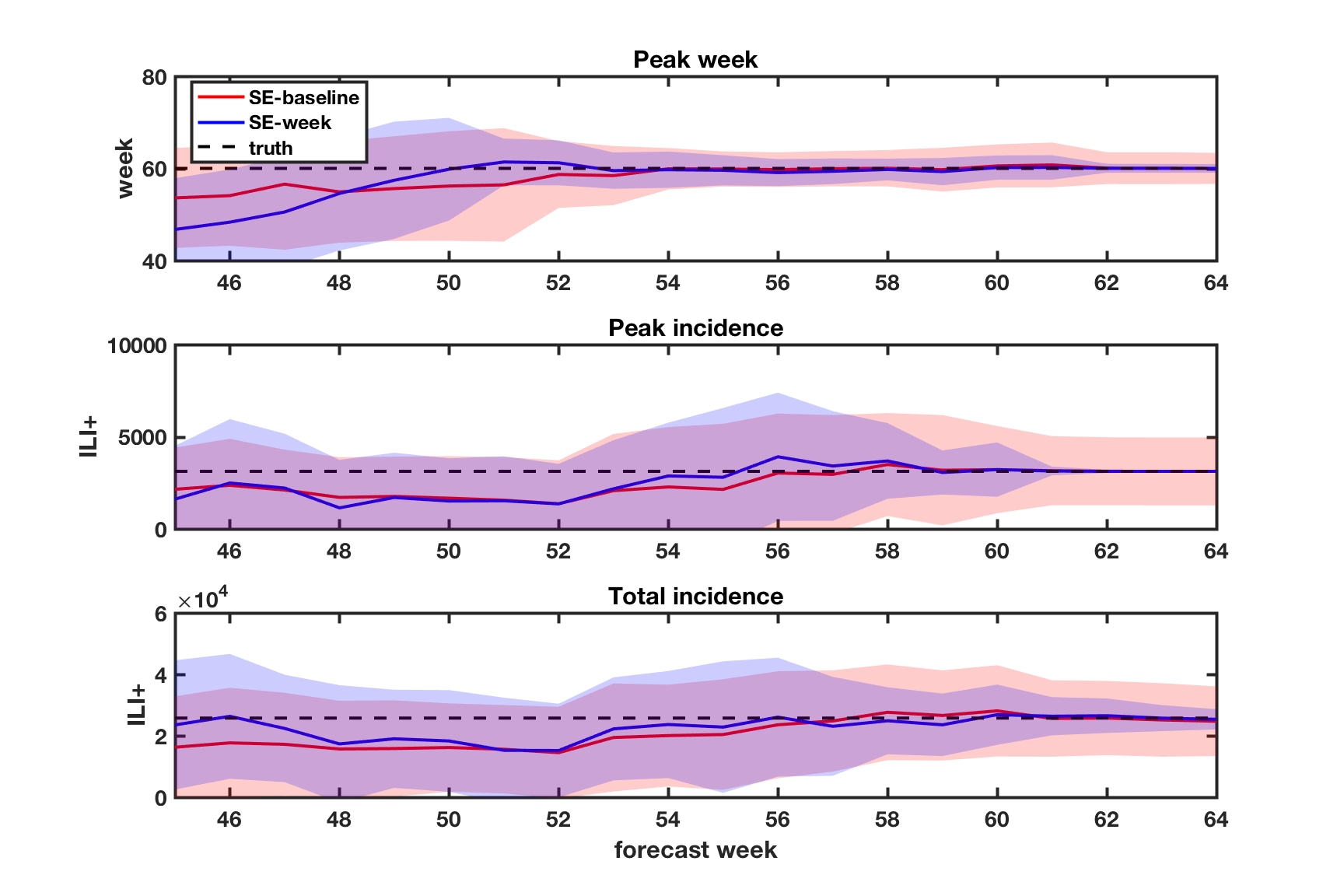

Supplement: S2 Fig — The weekly SE-baseline and SE-week forecasts are shown for a sample outbreak. 95% credible intervals are indicated by the shaded areas. (TIF) [file pcbi.1005801.s003.tif]

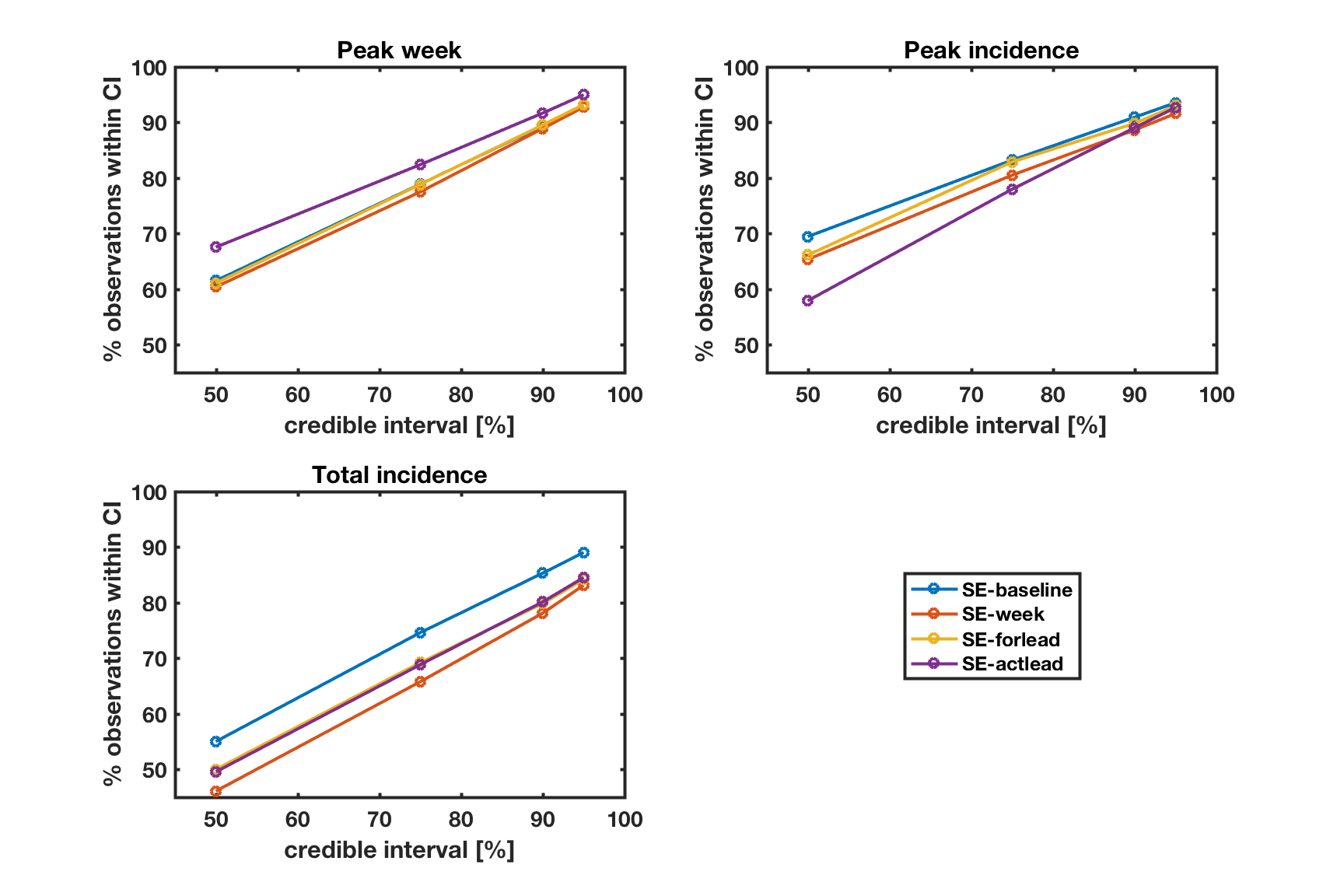

Supplement: S3 Fig — The points on the graph show the percent of observations falling within the specified credible intervals of the superensemble forecasts. (TIF) [file pcbi.1005801.s004.tif]

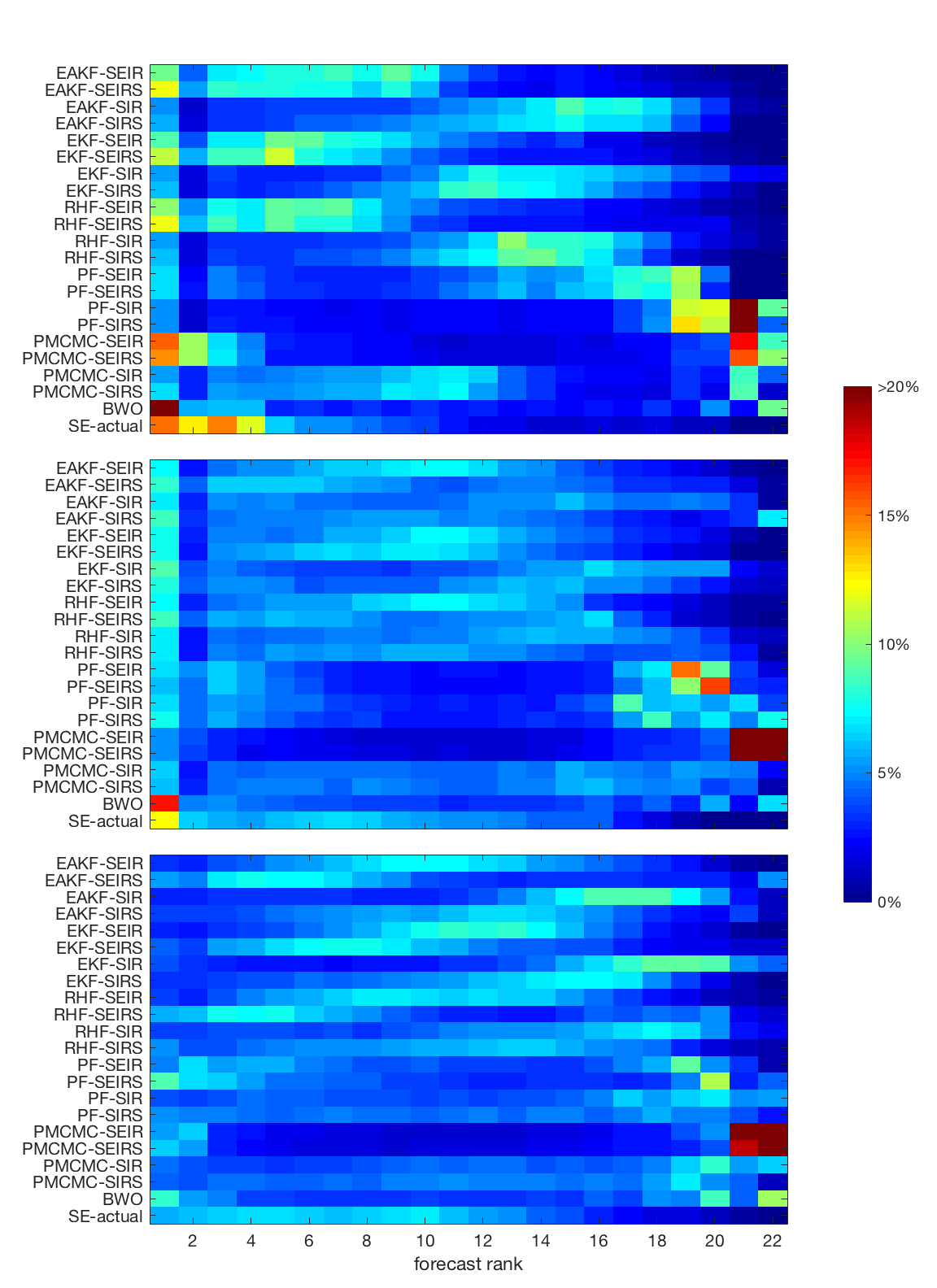

Supplement: S4 Fig — Same as Fig 6 in main text of paper, but with superensemble weights stratified by lead time of forecast relative to observed outbreak peak. (TIF) [file pcbi.1005801.s005.tif]
